# Investigating the causal association between gut microbiota and type 2 diabetes: a meta-analysis and Mendelian randomization

**DOI:** 10.3389/fpubh.2024.1342313

**Published:** 2024-06-19

**Authors:** Ting Liu, Yang Cao, Ning Liang, Xiaoqi Ma, Jing-ai Fang, Xiaodong Zhang

**Affiliations:** ^1^Department of Nephrology, The First Hospital of Shanxi Medical University, Taiyuan, China; ^2^Department of Nephrology, Shanxi Medical University, Taiyuan, China

**Keywords:** T2DM, meta-analysis, two-sample MR analysis, multi-sample MR analysis, MR-BMA gut microbiota, MR-BMA

## Abstract

**Background:**

Studies have shown that gut dysbiosis contributes to the pathophysiology of type 2 diabetes mellitus (T2DM). Identifying specific gut microbiota dysbiosis may provide insight into the pathogenesis of T2DM.

**Purpose:**

This study investigated the causal relationship between gut microbiota and T2DM using meta-analysis and Mendelian randomization (MR).

**Methods:**

In the first part, we searched for literature on gut microbiota and T2DM, and conducted a meta-analysis. We observed differences in glycosylated hemoglobin and fasting blood glucose levels in both groups. Second, we obtained GWAS data from genome-wide association study database 19 (GWAS). We used two-sample MR analysis to verify the forward and reverse causal associations between gut microbiota and T2DM. Additionally, we selected the European GWAS data from the European Bioinformatics Institute (EBI) as a validation set for external validation of the MR analysis. In the third part, we aimed to clarify which gut microbiota contribute to the degree of causal association between group disorders and T2DM through multivariate MR analysis and Bayesian model averaging (MR-BMA).

**Results:**

1. According to the meta-analysis results, the glycated hemoglobin concentration in the gut probiotic intervention group was significantly lower than in the control group. Following treatment, fasting blood glucose levels in the intervention group were significantly lower than those in the control group. 2. The results of two samples MR analysis revealed that there were causal relationships between six gut microbiota and T2DM. *Genus Haemophilus* and *order Pasteurellaceae* were negatively correlated with T2DM. *Genus Actinomycetes, class Melanobacteria* and *genus Lactobacillus* were positively correlated. Reverse MR analysis demonstrated that T2DM and gut microbiota did not have any reverse causal relationship. The external validation data set showed a causal relationship between gut microbiota and T2DM. 3. Multivariate MR analysis and MR-BMA results showed that the independent *genus Haemophilus* collection had the largest PP.

**Conclusion:**

Our research results suggest that gut microbiota is closely related to T2DM pathogenesis. The results of further MR research and an analysis of the prediction model indicate that a variety of gut microbiota disorders, including *genus Haemophilus*, are causally related to the development of T2DM. The findings of this study may provide some insight into the diagnosis and treatment of T2DM.

**Systematic review registration:**

https://www.crd.york.ac.uk/PROSPERO

## 1 Introduction

T2DM is one of the most important diseases affecting the health of the global population (1, 2). It will be beneficial to improve the prognosis of patients with T2DM by actively exploring the pathogenesis of the disease. Numerous factors contribute to the development of T2DM, including insulin resistance, insulin secretion defects, changes in the composition of cell membrane lipids, inflammation, gastrogut complications, viral infections, and disturbances in gut microbiota (3–5), among which gut microbiota disorders are a hot topic and target of current research on the pathogenesis of T2DM (6).

Recent studies have shown gut microbiota structure and function changes may participate in T2DM pathogenesis through the “pancreas gut axis” (7–9). The gut microbiota consists of microorganisms that colonize the human intestines. Typically, the gut microbiota consists of anaerobic bacteria and can be classified into six phyla: *Firmicutes (Lactobacillus, Enterococcus, Clostridium), Bacteroidetes, Proteobacteria (Enterobacteria), Actinomycetes (Bifidobacterium), Fusobacteria*, and *Verrucococcus*. Of the total gut microbiota, 64%, 23%, 8%, and 3% come from the first four phyla (10). There are several physiological functions performed by gut bacteria and their metabolites, including maintaining the host's gut microecological balance, enhancing immunity, regulating gut motility, affecting nutrition absorption, regulating glucose through gut hormone secretion and activating immunity, and regulating fat metabolism (11–13).

Clinical studies have shown that T2DM patients' microbiota differs significantly from healthy individuals. In T2DM patients, *Clostridium* and *Firmicum* were significantly lower than in healthy individuals. It was found, however, that the ratio of Bacteroides to *Escherichia coli* increased with the decrease of glucose tolerance in the random subjects, which supported the hypothesis that there was an imbalance in the gut microbiota during the onset and development of T2DM (14, 15). Compared to non-T2DM patients, T2DM patients have a higher level of LPS expression. There is evidence that an imbalance of gut microbiota may promote the expression of pro-inflammatory factors such as LPS and inhibit the expression of anti-inflammatory factors, thereby causing inflammation and worsening T2DM (16–19).

Furthermore, the blood sugar concentration of patients with T2DM decreased significantly after taking *lactic acid bacteria*, indicating that gut microbiota improvement affects blood sugar changes positively. A significant increase in glutathione oxidase and dismutase activity was found in the blood, suggesting gut microbiota improvement affects blood sugar levels. It should be noted that most of the studies mentioned above are clinical cross-sectional studies and cohort studies. Several limitations associated with observational studies make it difficult to establish a causal link between changes in gut microbiota and T2DM.

As an emerging causal inference method, MR analyses GWAS utilizing genetic variation as instrumental variables (IVs) to investigate causal relationships between risk factors and outcomes (20–22). The research process is similar to that of a clinical RCT. The method is widely used in epidemiological causal association studies. In recent years, MR research has made significant advancements. As a result, it positively affects identifying the causal relationship between diseases. However, relatively few studies have focused on the relationship between gut microbiota and T2DM pathogenesis. Consequently, there is no consensus regarding whether gut microbiota contributes to T2DM risk. In a two-sample MR study, Xiang et al. found no evidence that 28 species of gut microbiota were associated with the risk of developing T2DM. According to their study, *Streptococcus and Acidaminococcaceae* were likely associated with a borderline positive correlation with T2DM risk (23).

In this study, we used meta-analysis to identify a clinical correlation between gut microbiota and T2DM. Then, we used MR research methods to develop a predictive model of gut microbiota for T2DM. The study identified a causal link between gut bacteria and T2DM risk using genetic evidence.

## 2 Materials and methods

**Figure 1:**
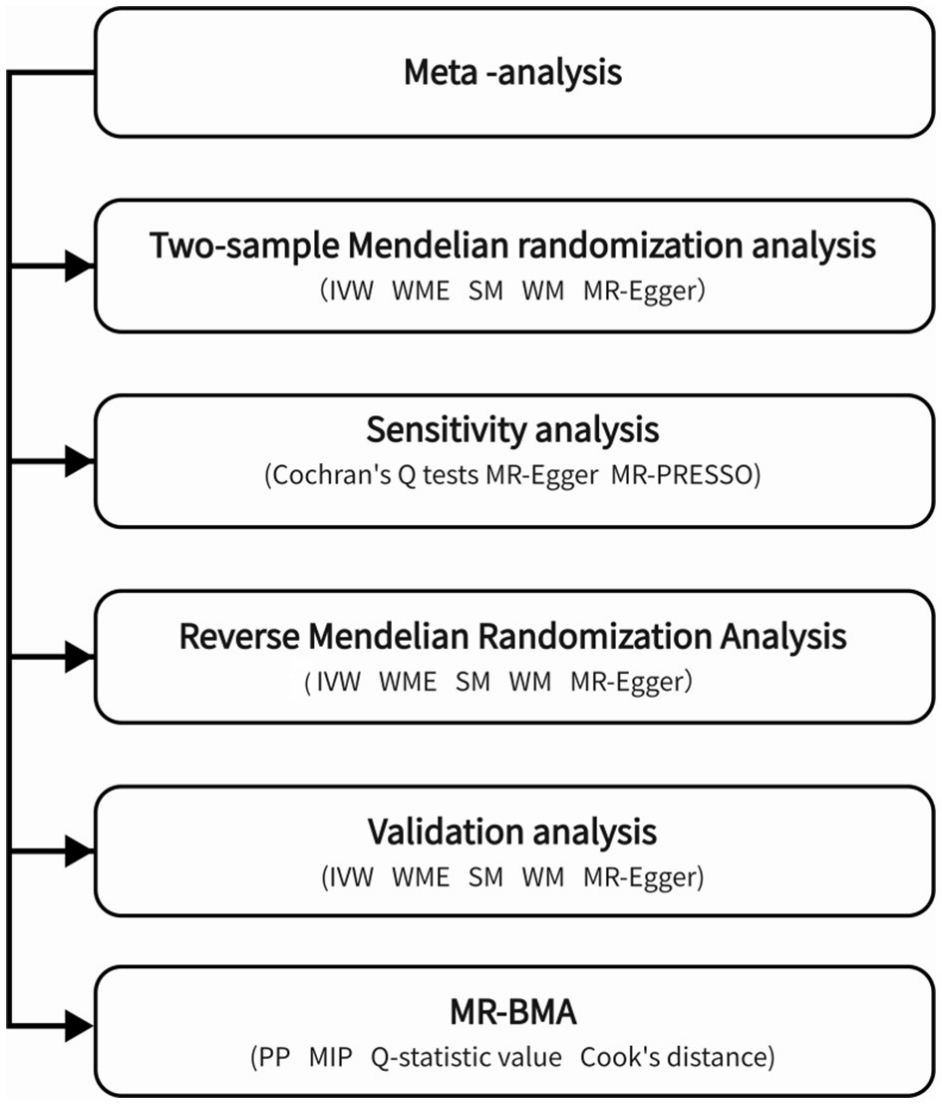
This figure illustrates a flow chart.

There are three stages to this research, as shown in Figure 1.

### 2.1 Meta-analysis

Using the subject headings and free words “gut microorganisms,” “type 2 diabetes,” and “clinical randomized controlled trials,” we searched PubMed, Web of Science, Cochrane Library, and Embase databases, respectively. The retrieved documents were not restricted to countries. The language is English, and the period is July 2023.

Inclusion criteria: the study population is T2DM; the research type is a clinical randomized controlled trial; the intervention measures are gut probiotics or similar preparations; and the outcome indicators are fasting blood glucose and glycated hemoglobin levels.

Exclusion criteria: exclude case reports utilizing animal and cell models; literature that has been repeatedly included; literature with incomplete data; unpublished literature; literature that does not include T2DM in the study population; or literature that does not include probiotics or similar preparations in the intervention.

Data extraction and quality evaluation: we imported the literature from four databases into the title list. Using a double-masked method, two investigators screened the literature. They extracted data based on study inclusion and exclusion criteria, including author, year, country, age, sample size, intervention, and outcome measures. We assessed bias risk in randomized controlled trials using a tool developed by the Cochrane Collaboration.

This study was conducted by the Preferred Reporting Items for Systematic Reviews and Meta- Analyses (PRISMA) guidelines. We assessed bias risk in randomized controlled trials using the bias risk assessment tool developed by the Cochrane Collaboration. The PROSPERO registration number was CRD42023444465.

### 2.2 Data source

We obtained T2DM and gut microbiota data from the public GWAS database (https://gwas.mrcieu.ac.uk). All data used in this study were publicly available GWAS summary results. The research does not involve ethical or privacy issues; as a result, an ethical review is unnecessary.

This study utilized gut microbial GWAS data from the MiBioGen consortium (24, 25). This study included genome-wide association and gut microbial metagenomic data from 18,340 European populations in 24 cohorts. Genomics-based association studies could be used to examine the genetic association between the relative abundance of gut microorganisms and the genes of the human host. In addition, this study included 271 gut microorganisms for further analysis.

T2DM data from the UK Biobank (UKBB) were used in this study, containing 10,894,596 SNPs in 337,159 individuals, including 335,026 in the control group and 2,133 in the experimental group. In addition, 655,666 Europeans were selected for verification from EBI, including 654,488 experimental subjects and 1,178 control subjects. The dataset contains 5,030,727 SNPs.

### 2.3 MR analysis

The genetic variation selected as an IVs must meet the following three assumptions (26): (1) Correlation assumption: The selected IVs must have a close relationship with T2DM. (2) Independence assumption: IV should not be confounded by confounding factors; (3) Exclusion restriction assumption: IV can only influence T2DM through the gut microbiota, not other channels.

We selected genetic variant SNP sites with genome-wide significance (*P* < 1 × 10–5) for T2DM. Select SNPs based on the linkage disequilibrium parameter (r^2^) threshold of 0.01 and the genetic distance of 139 10,000 kb to guarantee their independence and eliminate the impact of linkage disequilibrium (LD). Our analysis followed the guidelines for conducting MR studies. This report followed the guidelines for Strengthening the Reporting of Studies with MR (STROBE-MR).

#### 2.3.1 Two-sample MR analysis

In this study, causal effects were estimated using five methods: inverse variance weighting (IVW), MR Egger, weighted median (WME), simple mode (SM), and weighted mode (WM). The IVW method assumes that all genetic variants contain valid IVs. The ratio method calculates the causal effect value of a single instrumental variable. Each estimate is aggregated for weighted linear regression to obtain the total effect value. It is important to note that the MR-Egger method differs from the IVW method in that an intercept term is considered in the regression. The WME method takes advantage of the intermediate effects of all available genetic variants to get an estimate. This is accomplished by weighting the inverse variance of each SNP's association with the outcome. SM and WM are modality-based methods. Modality-based estimation models aggregate SNPs with similar causal effects and return estimates of causal effects for most cluster SNPs. According to WM, every SNP's impact on the cluster is weighted by the inverse variance of its effect (27). This study utilized the IVW method as its preferred method for causal effect estimation due to its higher testing efficiency than the other four MR methods. The results were visualized using forest maps and scatter plots.

#### 2.3.2 Inverse MR analysis

Using T2DM as the exposure variable and associated gut microbiota as the outcome variable, we examined whether T2DM has the exact causal relationship with the gut microbiota. In order to assess the causal relationship between the two diseases, MR analysis was conducted once again without modifying either the statistical method or the data source. This study aimed to determine if T2DM affects the gut microbiome. The statistical methods used at this stage are the same as those used at the previous stage.

#### 2.3.3 Validation analysis

Selecting 655,666 Europeans from EBI as the validation population, we performed MR analyses again to determine whether the gut microbiome was causally associated with developing different types of T2DM. At this stage, the statistical method remains consistent with the above.

#### 2.3.4 MR research based on Bayesian model

The multivariate MR analysis could simultaneously estimate the causal relationship between multiple risk factors for T2DM. In essence, it is an extension of unit MR analysis. Based on multivariate MR analysis, we used MR Bayesian model averaging (MR-BMA) probability to quantify the degree of causal association between different gut microbiomes and T2DM.

Based on the MR-BMA research method, we predicted the causal importance of the gut microbiota that was significantly related to T2DM and considered the potential pleiotropic effects. A posterior probability (PP) is assigned to each candidate model. After adding PP to each candidate risk factor, we calculated each biomarker's marginal inclusion probability (MIP) and reported each biomarker's model average causal effect (MACE) on T2DM. The best model was preferentially selected based on the PP value ranking of each model (threshold set to 0.02). Finally, we used Q statistics and Cook distance to identify outliers and strong influence points for model diagnosis. The MR-BMA analysis was repeated after these factors were removed.

### 2.4 Quality control

In this study, quality control was implemented on MR results that had a *P* < 0.05. The quality control process included sensitivity analysis, heterogeneity testing, and horizontal gene pleiotropy testing. Sensitivity analysis was conducted using the leave-one-out method by deleting individual SNPs in sequence and calculating the combined effect size of the remaining SNPs to evaluate the impact of each SNP on the results. The heterogeneity test uses the Cochran Q test to determine the heterogeneity of SNPs. It also evaluates the possibility of causal effect estimation bias due to SNP measurement errors resulting from differing analysis platforms, experimental conditions, and sample populations. In horizontal pleiotropy testing, the intercept term of MR-Egger regression was used to assess whether IVs impact outcomes through pathways other than exposure.

### 2.5 Statistical analysis

We completed Meta-analysis with RevMan 5.3 and STATA 16.0 statistical software, and we conducted MR analysis using R packages such as “Mendelian Randomization” and “Two Sample MR.” Furthermore, Multivariate MR analysis uses the R language code and functions related to MR-MBA publicly released on GitHub (https://github.com/verena-zuber/demo~AMD).

## 3 Results

### 3.1 Meta-analysis

**Figure 2:**
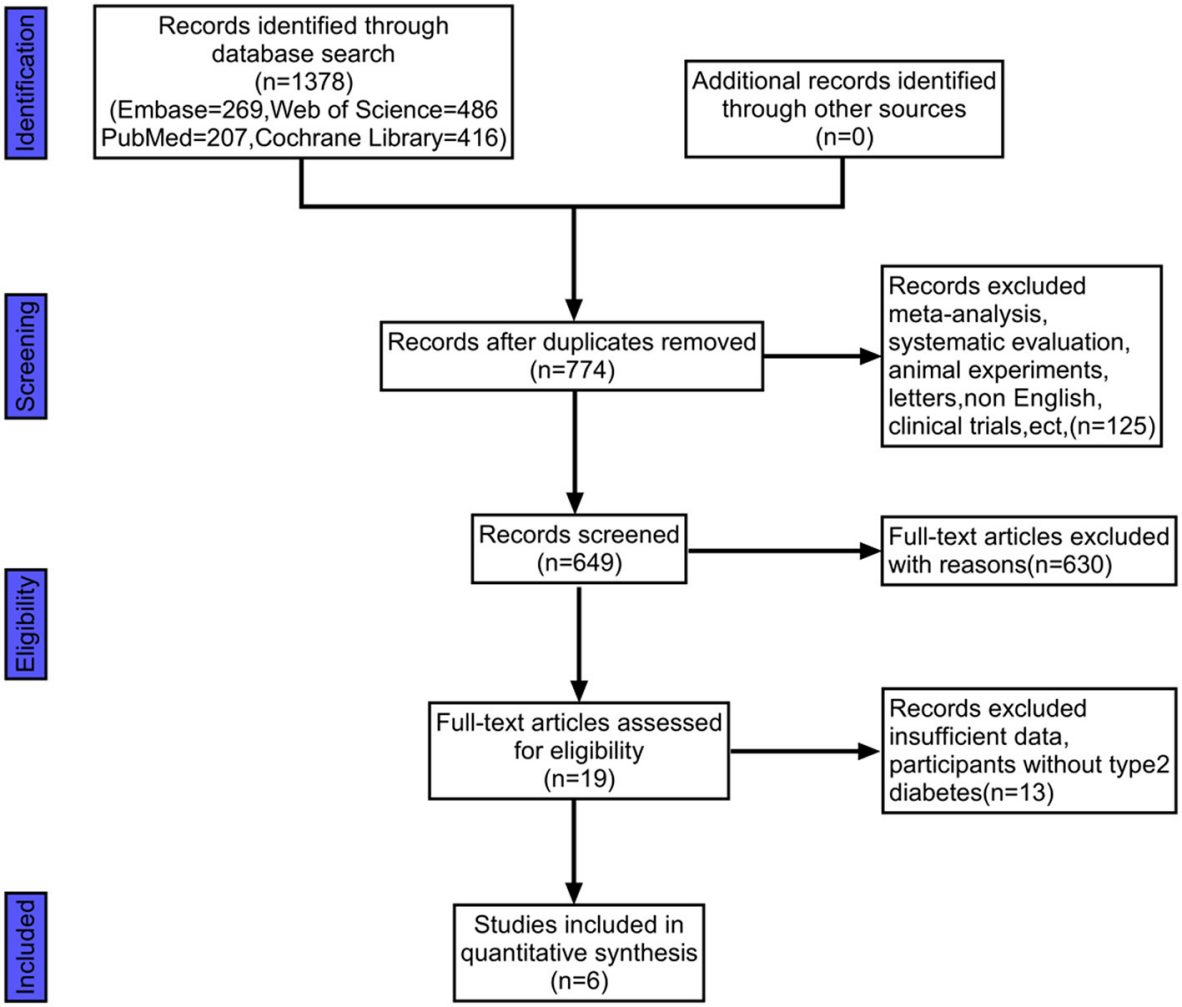
This figure illustrates the flow chart of literature screening.

We retrieved 1,378 documents from four databases: PubMed, Web of Science, Cochrane Library, and Embase. Ultimately, we included six documents after screening them according to inclusion and exclusion criteria. A flow chart of the screening process was shown in Figure 2. The six included in the literature Kanazawa et al. (28), Chaiyasut et al. (29), Khalili et al. (30), Rustanti et al. (31), Toejing et al. (32), Firouzi et al. (33) were all RCT trials, and their interventions included gut probiotics.

**Figure 3:**
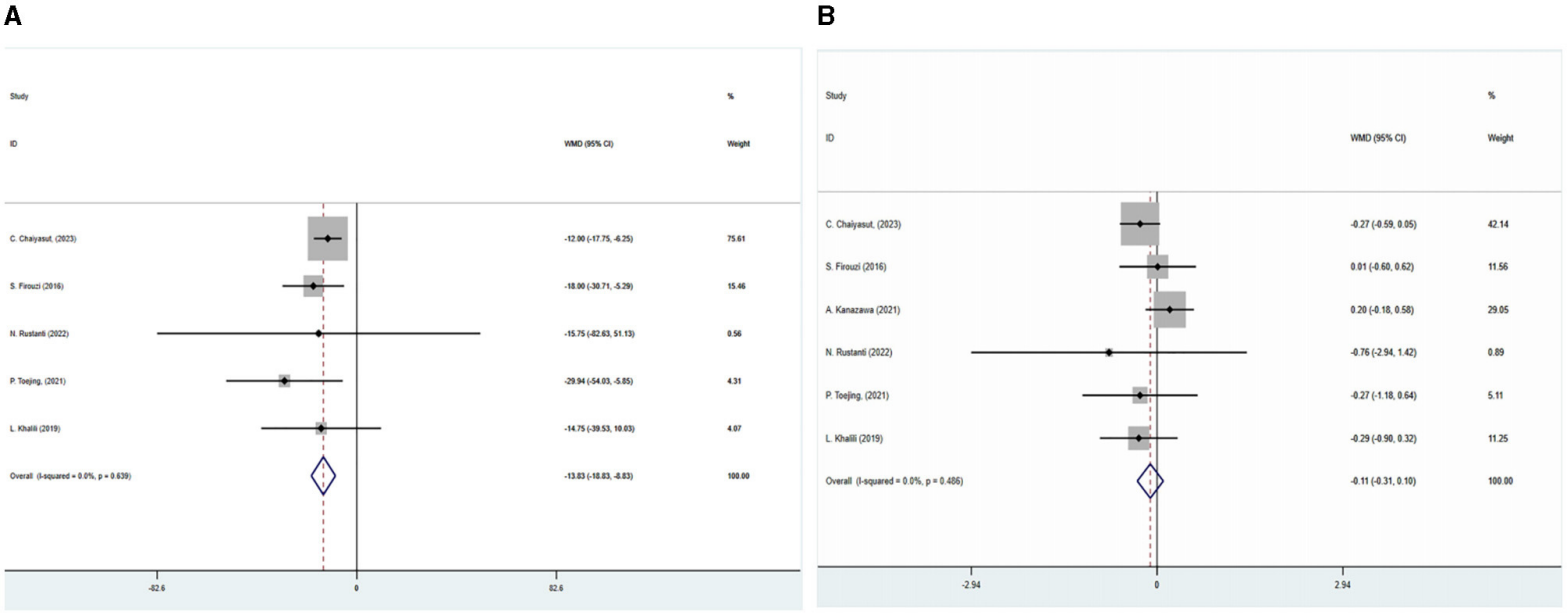
This figure displays a forest plot illustrating the impact of the combination of SGLT2 inhibitors with metformin on glycated, hemoglobin and fasting blood glucose levels were analyzed using a fixed-effects model, and the results were expressed as weighted mean difference (WMD) and 95% confidence interval (CI).

The outcome indicators of six of the included studies were glycated hemoglobin and fasting blood glucose. A meta-analysis using a fixed effects model demonstrated that the glycated hemoglobin of the intervention group was significantly lower than that of the control group after treatment (WMD = −0.108, 95%CI: −0.314–0.098, *P* = 0.305). There was no statistical heterogeneity among the studies (I^2^ = 0, *P* = 0.486). In the intervention group, fasting blood glucose levels were significantly lower than in the control group (WMD = −13.83, 95%CI: −18.831– −8.835, *P* < 0.05). Fasting blood glucose results were not statistically heterogeneous (I^2^ = 0, *P* = 0.639) (Figure 3). All studies were analyzed for sensitivity, and the results showed no significant differences. A publication bias analysis was also conducted using Begg's test: glycosylated hemoglobin (*P* = 0.647) and fasting blood glucose (*P* = 0.0229), indicating no publication bias present.

### 3.2 IVs screening

As exposures for the study, 271 gut microbes were selected from GWAS data on gut microbes involving 5,959 healthy Europeans. This study examined the relative abundance of 271 gut microorganism species, including 10 phyla, 16 classes, 22 orders, 48 families, 98 genera, and 77 species. A two-sample MR study was conducted using 271 species of gut microorganisms and 2,770 SNPs for the study, which was screened based on *P* < 1 × 10 – 5 and linkage disequilibrium thresholds.

### 3.3 Two-sample MR analysis and sensitivity analysis

Five TSMR methods were employed to analyze the causal relationship between the relative abundance of 271 species of gut microorganisms and T2DM, and the IVW method was primarily used to analyze the results.

Based on the IVW method, five gut microorganisms were associated with T2DM. These five types of gut microorganisms include three genera, one class and one order, each belonging to *genus Haemophilus* (OR = 0.997, 95%CI: −0.004–−0.002, *P* = 0.003), *genus Actinobacteria* (OR = 1.003, 95%CI: 0.002–0.005, *P* = 0.004), *order Pasteurellaceae* (OR = 0.998, 95%CI: −0.003–−0.001, *P* = 0.009), *class Melanobacteria* (OR = 1.001, 95%CI: 0.001–0.002, *P* = 0.034), *genus Lactobacillus* (OR = 1.001, 95%CI: −0.00001–0.002, *P* = 0.035).

**Figure 4:**
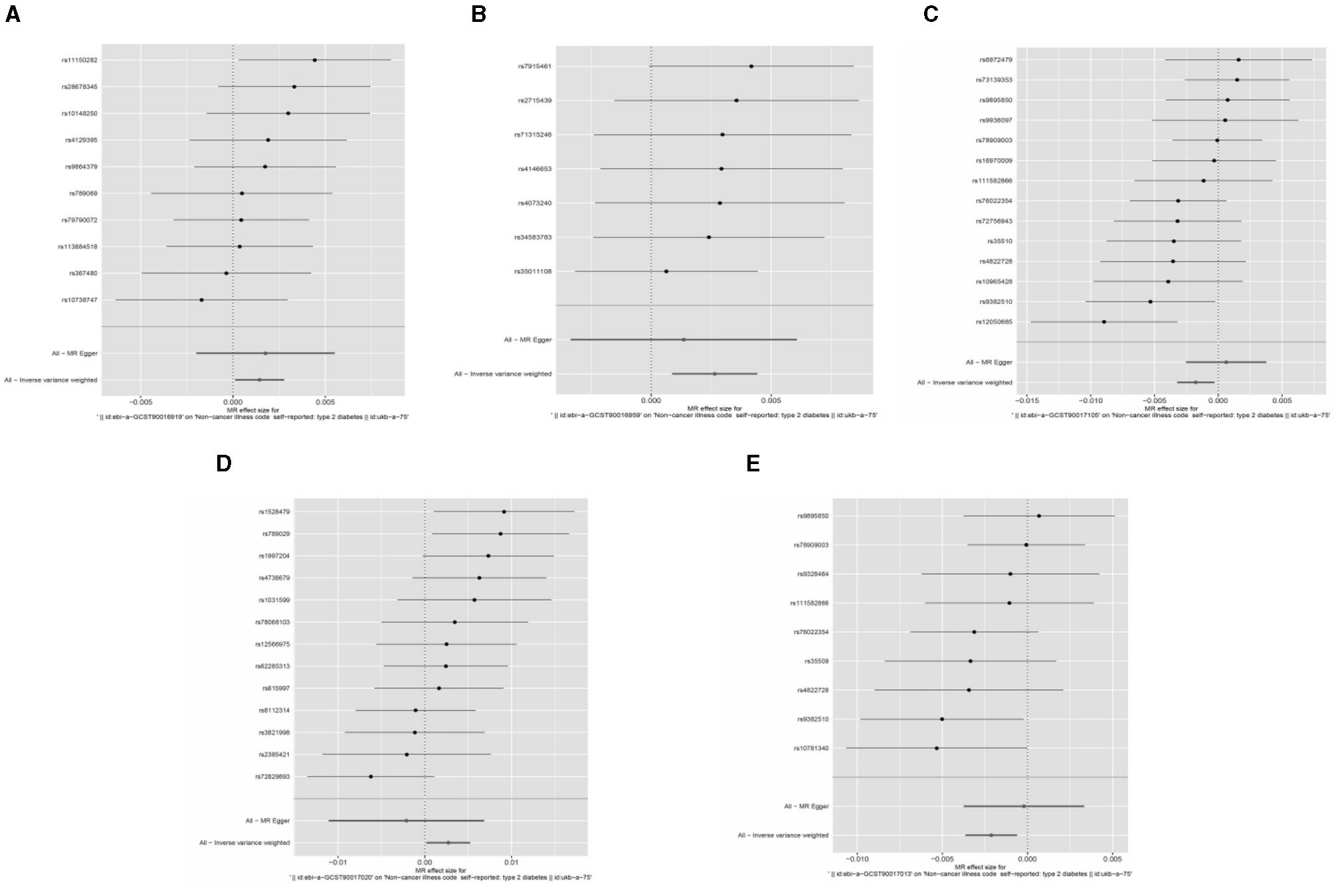
Forest map of Mendelian randomization analysis of gut microbes and T2DM.

**Figure 5:**
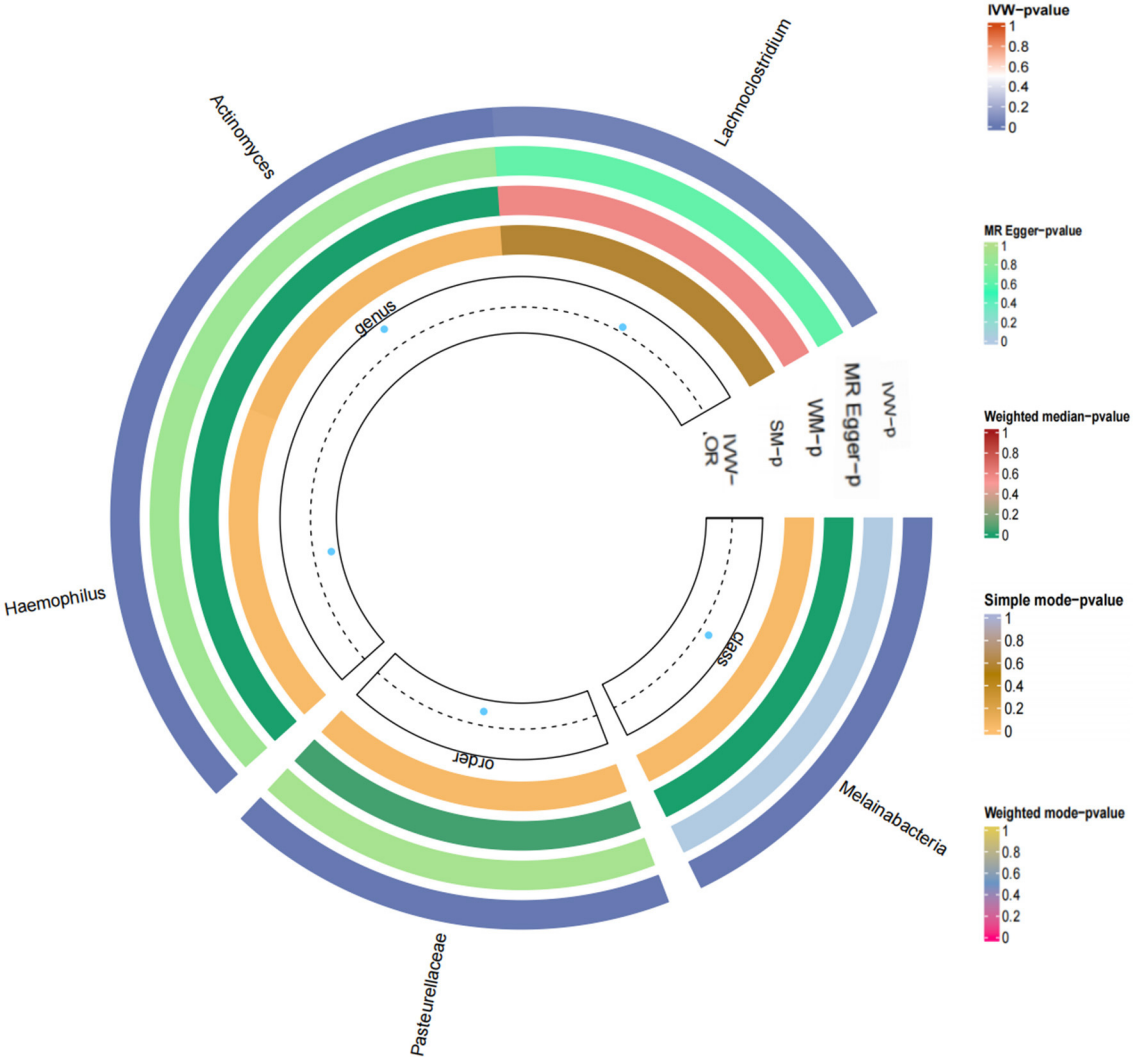
Loop diagram of the Mendelian randomization analysis of gut microbes and T2DM.

Based on the Q test results, it was determined that there was no heterogeneity among the included SNPs, of which the five gut microbiota were (*P* = 0.642, *P* = 0.942, *P* = 0.256, *P* = 0.700, *P* = 0.186). According to the MR-Egger regression intercept, no horizontal pleiotropy exists in the association between gut microbiota and T2DM. These were *genus Haemophilus* (MR-Egger intercept = −0.0003, *P* = 0.272), *genus Actinobacteria* (MR-Egger intercept = 0.0002, *P* = 0.588), *order Pasteurellaceae* (MR- Egger intercept = −0.0003, *P* = 0.112), *class Melanobacteria* (MR-Egger intercept = −0.00004, *P* = 0.864), *genus Lactobacillus* (MR-Egger intercept = 0.0003, *P* = 0.295) (Figures 4, 5). According to the MR results of the retention method, no SNPs significantly impacted the effect estimation of gut microbiota and T2DM. As a result, the causal relationship appeared stable (Table 1).

**Table 1:** Summary of the results of two-sample Mendelian randomization analysis of gut microbiota and T2DM.

**Gut microbiota**	**Traits**	**Method**	**OR**	**OR (95%CI)**	**Beta**	***p*-value**	**Heterogeneity (IVW)**		**MR-Egger regression**	
							**Cochran's Q**	***p*** **-value**	**Egger-intercept**	***p*** **-value**
*Genus Haemophilus*	T2DM	MR Egger	1.000226	0.997122–1.003339	−0.0003	0.871954	6.955836	0.641718	−0.00025	0.271674
		WME	0.996749	0.995187–0.998313	−0.00233	0.03201				
		IVW	0.99732	0.996157–0.998484	−0.00223	0.073305				
		SM	0.996111	0.992895–0.999337	−0.00346	0.073305				
		WM	0.996252	0.99315–0.999362	−0.0033	0.074944				
*Genus Actinomyces*	T2DM	MR Egger	1.00036	0.996372–1.004363	0.001358	0.597155	1.734946	0.942392	0.000151	0.588458
		WME	1.003139	1.001418–1.004863	0.002902	0.012449				
		IVW	1.003343	1.0020 14–1.0046 74	0.002646	0.003522				
		SM	1.003 311	1.000101–1.006532	0.002 935	0.141 957				
		WM	1.003356	1.00026–1.006462	0.00291	0.115516				
*Order Pasteurellales*	T2DM	MR Egger	1.132908	0.759943–1.688918	0.000542	0.733494	17.00006	0.256174	−0.00031	0.111692
		WME	1.076112	0.961559–1.204312	−0.00107	0.244946				
		IVW	1.020298	0.908714–1.145584	−0.00186	0.008605				
		SM	1.168823	0.954992–1.430534	−0.00328	0.079287				
		WM	1.163325	0.968436–1.397433	7.33E−05	0.961784				
*Class Melainabacteria*	T2DM	MR Egger	1.010609	0.918467–1.111995	0.001758	0.385119	6.395291	0.699796	−0.00003	0.864495
		WME	0.985414	0.954629–1.017191	0.000948	0.287669				
		IVW	0.983894	0.959877–1.008511	0.001443	0.033665				
		SM	0.985494	0.928972–1.045454	0.000611	0.694069				
		WM	0.985966	0.922925–1.053312	0.00068	0.624704				
*Genus Lachnoclostridium*	T2DM	MR Egger	1.597226	1.182501–2.157402	−0.00212	0.652128	16.11211	0.186155	0.000327	0.294713
		WME	1.128202	1.081772–1.176625	0.002463	0.129611				
		IVW	1.127492	1.091355–1.164826	0.002714	0.035294				
		SM	1.171256	1.053803–1.3018	0.002973	0.331111				
		WM	1.166773	1.054457–1.291053	0.002144	0.469648				

OR, odds ratio; IVW, inverse variance weighted method; WM, weighted mode; WME, weighted median method; SM, simple mode; T2DM, type 2 diabetes mellitus.

### 3.4 Inverse MR analysis

**Figure 6:**
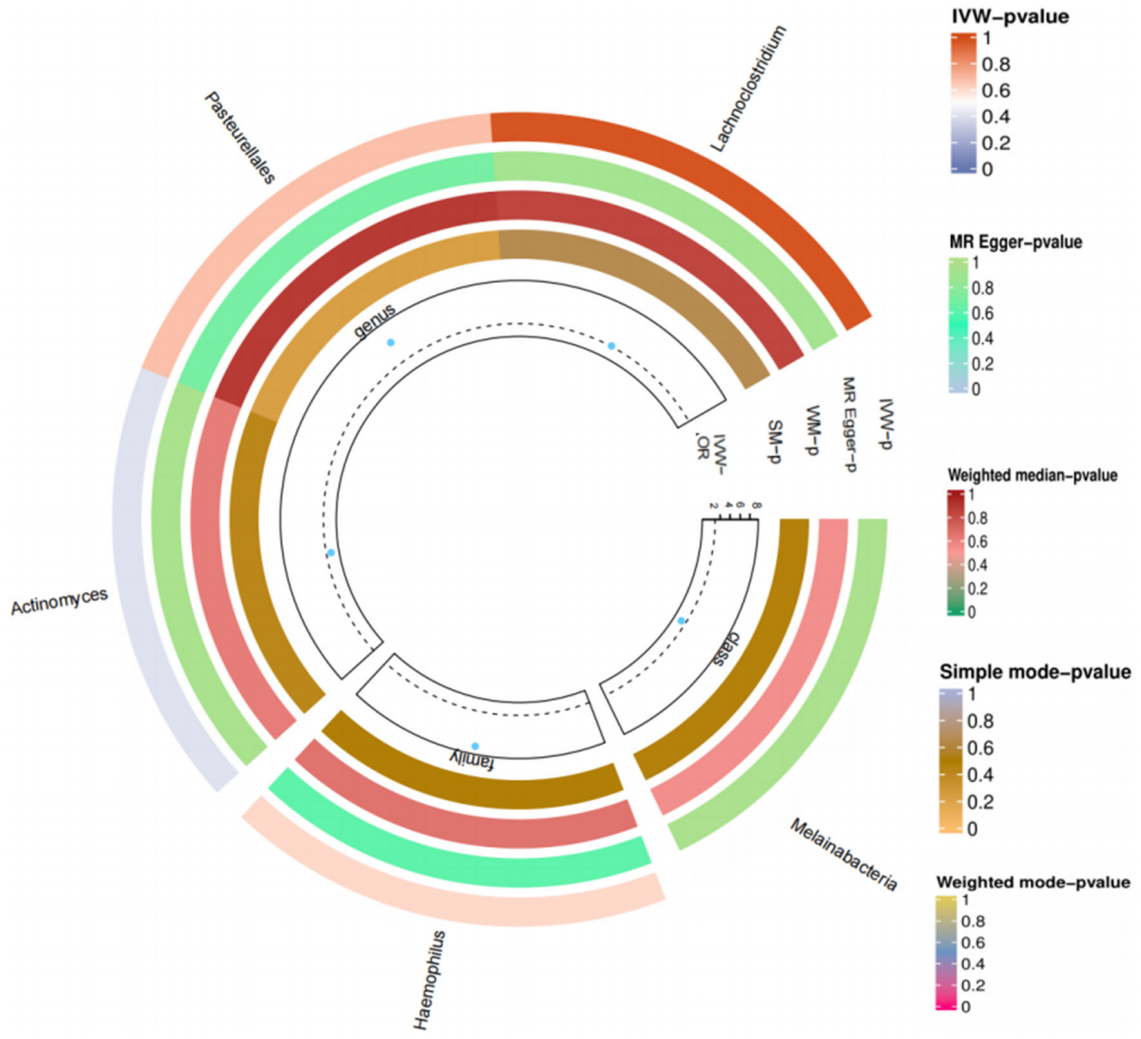
Loop diagram of the gut microbiome and T2DM in reverse Mendelian randomized analysis.

We again conducted a two-sample Mendelian randomized study; T2DM was used as an exposure factor, and six related gut microorganisms were selected as outcome factors. The results indicated no causal link between T2DM and gut microbiota. *Genus Haemophilus* (OR = 5.555, 95%CI: 0.002–17,959.04, *P* = 0.678), *genus Actinobacteria* (OR = 0.021, 95%CI: 2.74E-06–160.7864, *P* = 0.397), *order Pasteurellaceae* (OR = 8.029, 95%CI: 0.003–2,292.23, *P* = 0.608), *class Melanobacteria* (OR = 0.031, 95%CI: 1.33E-06–706.659, *P* = 0.497), *genus Lactobacillus* (OR = 1.112, 95%CI: 0.001–1026.964, *P* = 0.976). The study suggests that T2DM and gut microbiota do not have any causal relationship (Figure 6).

### 3.5 Verification analysis

As the validation population for the Two-Sample MR analysis verification, 655,666 European individuals were selected from EBI. The results of the IVW method showed that 223 gut microorganisms are causally related to T2DM. We used the Q test to exclude heterogeneity and the MR-Egger regression method to exclude gut microorganisms that exhibit horizontal pleiotropy.

**Figure 7:**
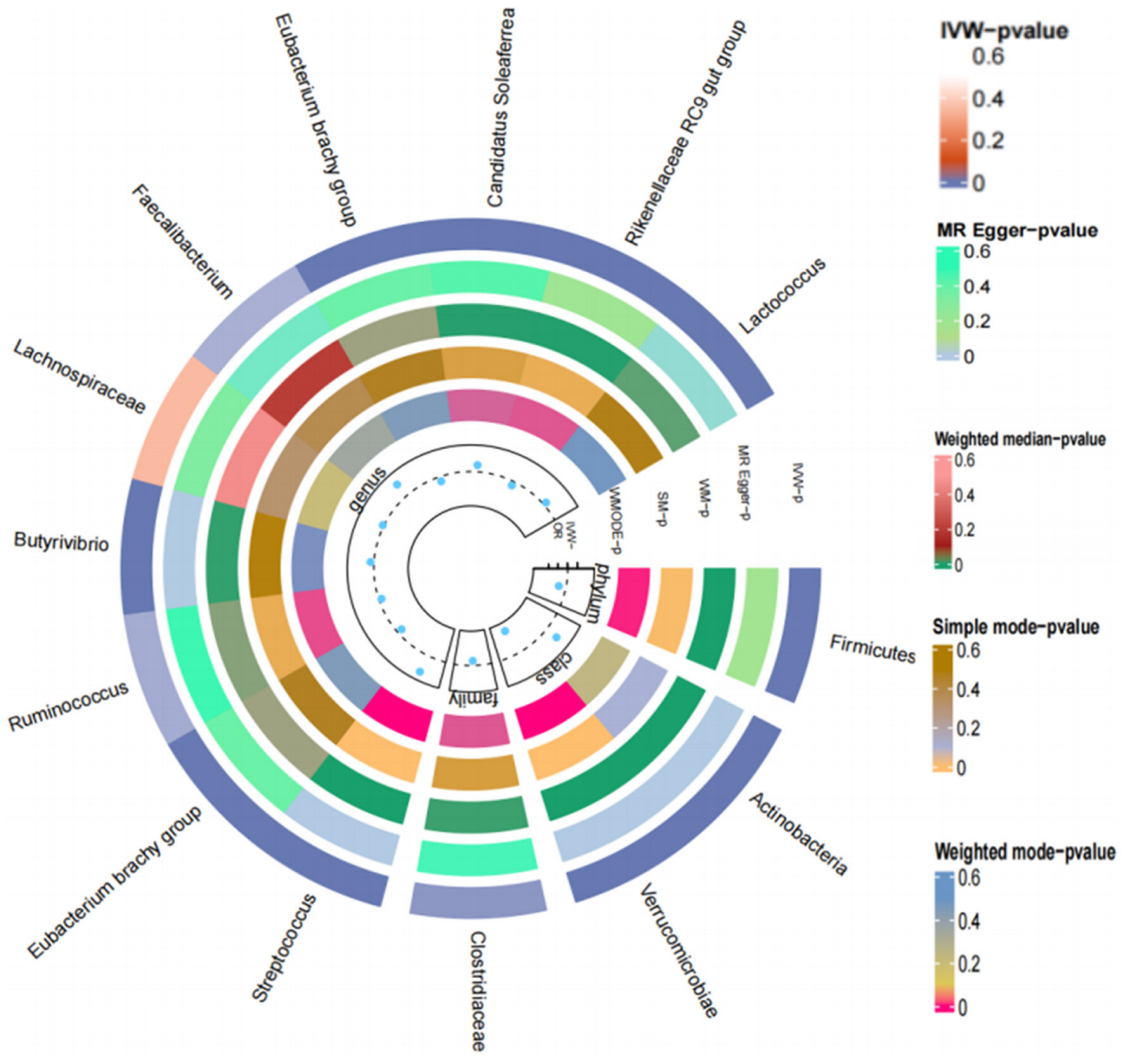
Loop diagram of the Mendelian randomization validation analysis of gut microbes and T2DM.

Ultimately, 22 types of gut microorganisms were found. These included *genus Streptococcus* (*P* = 1.08E-60), *phylum Actinobacteria* (*P* = 1.77E-16), *genus Eubacterium* (*P* = 4.04E-14), *genus Ruminococcus* (*P* = 1.42E-10), *genus VerrucomicrobiaUCG003* (*P* = 1.57E-09) and *UCG010* (*P* = 2.51E-09), *family Clostridiaceae* (*P* = 9.61E-09), *genus Butyrivibrio* (*P* = 2.57E-07), *genus Lachnospira* (*P* = 4.23E-07), *genus Faecalibacterium* (*P* = 2.01E-06), *Eubacteriumredox* (*P* = 2.67E-06), *genus Candida* (*P* = 1.76E-05), *genus Ruminococcus* (*P* = 7.68E-05), *genus Thielella* (*P* = 0.0001), *genus Anaerobacteria* (*P* = 0.0003), *genus Rikenella* (*P* = 0.0007), *family Christeniaceae* (*P* = 0.0010), *genus Eisenbergella* (*P* = 0.0024), *genus Lactococcus* (*P* = 0.0026), *phylum Firmicutes* (*P* = 0.0043) and two unknown bacterial groups (*P* = 3.84E-10, *P* = 9.61E-09). According to the results, certain gut microbiota might be responsible for T2DM (Figure 7).

### 3.6 MR research based on Bayesian model

We performed MR-BMA analysis on six types of gut microbiota and included 55 SNPs in the analysis. Fourteen SNPs were deleted because the Cook distance exceeded 10, three SNPs were removed because the Q statistic exceeded the threshold, and 38 SNPs were retained and included in the model. Based on the MR-BMA model, the six gut microbiota were organized and combined into ten groups. We performed a multivariate MR analysis using weighted regression for each group of gut microbiota. There were six groups with PP > 0.02, and the optimal model of the *genus Haemophilus* had the highest (PP = 0.447).

Furthermore, we identified other optimal models (PP > 0.02) for the *genus Lactobacillus, genus Actinomycetes, order Pasteurellaceae, and class Melanobacteria*. The PP was then added up for each candidate's risk factor. The MIP of a risk factor represents the probability that it will be a causal determinant of disease risk, which was the risk factor with the high levels of causal evidence (MIP = 0.449), and the specific value was shown in Table 2.

**Table 2:** Ranking of gut microbiota for T2DM using MR-BMA.

**Risk factor or model**	**MIP**	**Rank by MIP**	**MACE**	**PP**	**Rank by PP**	**Causal estimates**	***p***
**Model averaging employing 55 SNPs**							
*Class Melainabacteria*	0.372	1	0	0.372	1	−0.001	0.069
*Genus Lachnoclostridium*	0.134	4	0	0.134	2	0	0.703
*Genus Actinomyces*	0.126	5	0	0.125	3	−0.001	0.683
*Order Pasteurellales*	0.136	2	0	0.125	4	0.001	0.574
*Genus Haemophilus*	0.108	6	0	0.107	6	0.001	0.703
**Model averaging employing 41 SNPs (SNPs with Q-statistics** > **10 are not included)**							
*Order Pasteurelales*	0.254	2	0	0.235	2	0.001	0.069
*Genus Haemophilus*	0.227	3	0	0.226	3	0.001	0.050
*Genus Lachnoclostridium*	0.111	4	0	0.111	4	−0.001	0.901
*Genus Actinomyces*	0.1	5	0	0.099	5	−0.001	0.812
*Class Melainabacteria*	0.075	6	0	0.074	6	−0.001	0.752
**Model averaging employing 38 SNPs (SNPS that exceed the Cook's distance threshold are excluded)**							
*Genus Actinomyces*	0.449	1	−0.001	0.447	1	−0.002	0.069
*Genus Lachnoclostridium*	0.213	2	0	0.212	2	−0.002	0.604
*Genus Haemophilus*	0.094	5	0	0.094	3	0.001	0.802
*Order Pasteurellales*	0.101	4	0	0.093	5	0.001	0.772
*Class Melainabacteria*	0.051	6	0	0.051	6	−0.001	0.911

SNPs, single nucleotide polymorphisms; MIP, marginal inclusion probability; MACE, model-average causal effect; MR-BMA, MR based on Bayesian model averaging; PP, posterior probability.

## 4 Discussion

In this study, we used meta-analysis to observe that T2DM patients who took gut probiotics had lower fasting blood sugar and glycated hemoglobin levels than the control group. Our meta-analysis suggests that regulating gut microbiota disorders may delay T2DM progression. It is unclear, however, which specific gut microbiota disorders maybe involved in T2DM pathogenesis. For this issue, we used MR research methods further to study the causal relationship between gut microbiota and T2DM. The results of the two-sample MR analysis showed that five gut microbiota are causally related to T2DM, among which the *genus Haemophilus* and *order Pasteurellaceae* were negatively correlated with T2DM; *genus Actinomycetes, class Melanobacteria*, and *genus Lactobacillus* were positively correlated. Reverse MR analysis demonstrated that T2DM and gut microbiota do not have any reverse causal relationship. Additionally, we selected data from EBI for external verification. MR analysis showed that 22 types of gut microbiota were causally related to T2DM. Based on the external validation data set, this study suggests a causal relationship between gut microbiota and T2DM.

Evidence shows gut microbiota structure and function changes are associated with T2DM (34). The pathophysiology of T2DM is influenced by gut microbiota and its related metabolites, including blood sugar metabolism, insulin resistance, and chronic inflammation. Recent studies have demonstrated an alteration in the gut microbiota of T2DM patients. Compared to the general population, T2DM patients had higher *genus Lactobacillus* (35)*, Enterococcus*, and *Clostridium* levels in the *phylum Firmicutes*. The *genus Bacteroidetes* was decreasing in the *phylum Bacteroides*. In the *phylum Actinobacteria*, the number of *genus Bifidobacteria* and *genus Rochella* decreases, and the gut microbiota changes signaling pathways that affect lipid and glucose metabolism, resulting in low-level inflammation, insulin resistance, and ultimately contributing to T2DM (15). Additionally, T2DM patients with better blood sugar control had a decrease in Enterobacteriaceae and Enterococcus compared with patients with poor blood sugar control. Furthermore, a significant improvement in insulin resistance was observed, with increased Bifidobacterium, Bacteroidetes, and bacteria that produced butyrate and propionate.

Generally, the mechanism by which gut microbiota disorder may contribute to T2DM includes three components (36): First, these gut microbiota can decrease the composition of short-chain fatty acid- producing bacteria in diabetic subjects, affect the metabolism of bile acids, and increase the microbiota in the gut tract (37). Second, they may prevent the recovery of the gut mucosal barrier, a process related to consuming potential gut pathogens and weakening digestion enzymes. As a result, it increases diabetes risk by promoting the production of trimethylamine nitrogen oxide (TMAO), which results in cholesterol accumulation and insulin resistance (38). Furthermore, these gut microbiotas can affect the progression of diabetes as well as other chronic diseases, such as systemic lupus erythematosus (39), rheumatoid arthritis (40), inflammatory bowel disease (41), chronic kidney disease (42), and ischemic stroke (43).

Numerous studies have demonstrated a close relationship between changes in gut microbiota structure and function and T2DM. However, given the diversity of gut microbiota, it is still unclear what type of gut microbiota disorder contributes to T2DM development. Previously, Xiang et al. (23) used MR research to demonstrate that *Streptococcus* and *Acidaminococcaceae* may have a critical positive correlation with T2DM risk. Our validation data set also revealed a positive association between *Streptococcus* and T2DM, consistent with Xiang et al.'s findings. Based on the research of Xiang et al., we expanded the types of gut microbiota and ranked the exposure factors. According to further MR study results, *genus Haemophilus* and *order Pasteurellaceae* were negatively correlated with T2DM and might play a protective role; *genus Actinomycetes, class Melanobacteria*, and *genus Lactobacillus* were positively correlated with T2DM, and they might be associated with T2DM. Additionally, the MR-BMA predictions suggested that the *genus Haemophilus* and T2DM had a significant causal relationship.

Some studies have demonstrated a close relationship between the *genus Haemophilus* and T2DM (44, 45). As stated by Nuli et al. (46), people with impaired glucose tolerance would have a higher proportion of *order Pasteurellaceae* and *genus Haemophilus* than those with normal glucose tolerance. Nevertheless, Zhang et al. (47) and Zhong et al. (48) found a reduction in the proportion of *genus Haemophilus* in new-onset diabetes. Studies have shown (49) that decreasing the abundance of *genus Haemophilus* in the intestine may produce short-chain fatty acids (SCFAs), such as butyric acid, propionic acid, and acetic acid, which may increase gut permeability (50). Several inflammatory factors, including lipopolysaccharides (LPS) and chyle particles, could pass through the gut epithelial barrier and activate downstream inflammatory pathways and pro-inflammatory cytokine cascades, resulting in a chronic systemic inflammatory response, impaired glucose metabolism, increasing insulin resistance, and eventually contributing to diabetes. In addition, A decrease in the number of *genus Haemophilus* species in the gut tract could also increase the production of uremic toxins (51, 52), including indenyl sulfate and p-cresol sulfate, which might lead to mitochondrial dysfunction, podocyte damage, thickening of the glomerular basement membrane, and complications such as diabetes kidney disease and coronary heart disease.

The results of our MR study suggest that the *genus Actinobacteria* and *genus Lactobacillus* may contribute to the progression of diabetes. The relevant research indicated that *genus Actinomycetes* and *genus Lactobacilli* in the intestine are associated with chronic inflammatory diseases, including T2DM. According to recent research (53–55), patients with T2DM have reduced amounts and proportions of bacteria from the *genus Actinobacteria* in their gut microbiota. In addition, the *genus Lactobacilli* positively correlates with fasting blood glucose and HbA1c levels. Several studies have shown that *genus Lactobacilli* levels were significantly higher among type 2 diabetics than healthy individuals (56, 57). Interestingly, we observed that *order Pasteurellaceae* and *class Melanobacteria* were associated with T2DM. More clinical and basic research is needed to determine the specific pathogenesis of these gut microbiota and T2DM.

This study has several advantages: First, it includes clinical and genetic studies. We used various research methods to elucidate gut microbiota and T2DM correlation. These researches included meta-analysis, two-sample MR analysis, and reverse validation with different data sets. In addition, we used MR-BMA torank the strength of the causal association between gut microbiota and T2DM. Second, we clarify the causal relationship between specific gut microbiota imbalances and T2DM. In this paper, we propose novel ideas and methods for studying the mechanism of the “pancreas-gut axis”.

Somethings could be improved in this study: Firstly, We could not further perform reverse MR analysis due to insufficient SNPs. The results of our study did not allow us to conclude that gut microbiota and T2DM were mutually related. Secondly, the MR results of the validation data set used in this article differ from those of the original data set. This may be due to the use of different consortium data. Lastly, although causal associations derived from MR studies have a certain amount of research value, they still require additional clinical and basic research confirmation. In light of this, we should be cautious when interpreting relevant research results.

## 5 Conclusion

Our research results suggest that gut microbiota is closely related to T2DM pathogenesis. The results of further MR research and an analysis of the prediction model indicate that a variety of gut microbiota disorders, including *genus Haemophilus*, are causally related to the development of T2DM. The findings of this study may provide some insight into the diagnosis and treatment of T2DM.

## Data availability statement

The original contributions presented in the study are included in the article/Supplementary material, further inquiries can be directed to the corresponding author.

## Author contributions

TL: Writing – review & editing. YC: Data curation, Software, Validation, Visualization, Writing – original draft. NL: Visualization, Writing – original draft. XM: Data curation, Formal analysis, Visualization, Writing – original draft. J-aF: Writing – review & editing. XZ: Conceptualization, Supervision, Writing – review & editing.
