# Supplementary material for: Investigating the causal association between gut microbiota and type 2 diabetes: a meta-analysis and Mendelian randomization
Source: Front Public Health. 2024 Jun 19;12:1342313. doi: 10.3389/fpubh.2024.1342313 (PMC11220316; doi:10.3389/fpubh.2024.1342313)
Supplement: Supplementary file 1 [file Table_1.DOCX]

library(TwoSampleMR)

setwd(" ")

ao<-available_outcomes()

write.csv(ao,"ao.csv")

ao<-read.csv("ao.csv")

bacteria=ao[grep("Gut microbiota abundance",ao$trait),]

bacteria$id

df_dat_exp<-extract_instruments(

outcomes = bacteria$id,p1=1e-5,clump = FALSE)

df_dat_exp$exposure

list_dat_exp<-split(df_dat_exp,list(df_dat_exp$exposure))

df_dat_exp_clump<-list()

for (i in 1:length(list_dat_exp)){

print(paste0("正在进行第_",i,"_个"))

time1=Sys.time()

df_dat_exp_clump[[i]]<-clump_data(list_dat_exp[[i]])

time2=Sys.time();print(time2-time1)

}

SNPS=do.call(rbind,df_dat_exp_clump)

SNPS=unique(SNPS$SNP)

SNPS

outcome_dat <-extract_outcome_data(snps=SNPS, outcomes="ukb-b-13806")

dat <- harmonise_data(exposure_dat = df_dat_exp,outcome_dat = outcome_dat)

res <- mr(dat)

res

write.csv(res,"res.csv")

OR <-generate_odds_ratios(res)

OR

write.csv(OR,"OR.csv")

ses_dat<-mr_heterogeneity(dat)

ses_dat

write.csv(ses_dat,"ses.csv")

pleiotropy<-mr_pleiotropy_test(dat)

write.csv(pleiotropy,"pleiotropy.csv")

mr_scatter_plot(res,dat)

res_single <- mr_singlesnp(dat)

mr_forest_plot(res_single)

mr_funnel_plot(res_single)
