# Supplementary material for: Investigating the causal association between gut microbiota and type 2 diabetes: a meta-analysis and Mendelian randomization
Source: Front Public Health. 2024 Jun 19;12:1342313. doi: 10.3389/fpubh.2024.1342313 (PMC11220316; doi:10.3389/fpubh.2024.1342313)
Supplement: Supplementary file 6 [file Table_6.DOCX]

("diabetes mellitus, type 2"[MeSH Terms] OR ("diabetes mellitus type 2"[Title/Abstract] OR "diabetes mellitus noninsulin dependent"[Title/Abstract] OR (("diabetes mellitus"[MeSH Terms] OR ("Diabetes"[All Fields] AND "Mellitus"[All Fields]) OR "diabetes mellitus"[All Fields]) AND "Ketosis-Resistant"[Title/Abstract]) OR (("diabetes mellitus"[MeSH Terms] OR ("Diabetes"[All Fields] AND "Mellitus"[All Fields]) OR "diabetes mellitus"[All Fields]) AND "Ketosis-Resistant"[Title/Abstract]) OR "ketosis resistant diabetes mellitus"[Title/Abstract] OR "diabetes mellitus non insulin dependent"[Title/Abstract] OR "diabetes mellitus non insulin dependent"[Title/Abstract] OR "non insulin dependent diabetes mellitus"[Title/Abstract] OR "diabetes mellitus stable"[Title/Abstract] OR "stable diabetes mellitus"[Title/Abstract] OR "diabetes mellitus type ii"[Title/Abstract] OR "NIDDM"[Title/Abstract] OR "diabetes mellitus noninsulin dependent"[Title/Abstract] OR "diabetes mellitus maturity onset"[Title/Abstract] OR "diabetes mellitus maturity onset"[Title/Abstract] OR "maturity onset diabetes mellitus"[Title/Abstract] OR "maturity onset diabetes mellitus"[Title/Abstract] OR "MODY"[Title/Abstract] OR "diabetes mellitus slow onset"[Title/Abstract] OR "diabetes mellitus slow onset"[Title/Abstract] OR ("Slow-Onset"[All Fields] AND "diabetes mellitus"[Title/Abstract]) OR "type 2 diabetes mellitus"[Title/Abstract] OR "noninsulin dependent diabetes mellitus"[Title/Abstract] OR "noninsulin dependent diabetes mellitus"[Title/Abstract] OR "maturity onset diabetes"[Title/Abstract] OR "diabetes maturity onset"[Title/Abstract] OR "maturity onset diabetes"[Title/Abstract] OR "type 2 diabetes"[Title/Abstract] OR "diabetes type 2"[Title/Abstract] OR "diabetes mellitus adult onset"[Title/Abstract] OR "adult onset diabetes mellitus"[Title/Abstract] OR "diabetes mellitus adult onset"[Title/Abstract])) AND ("Gastrointestinal Microbiome"[MeSH Terms] OR ("Gastrointestinal Microbiome"[Title/Abstract] OR "gastrointestinal microbiomes"[Title/Abstract] OR "microbiome gastrointestinal"[Title/Abstract] OR "gut microbiome"[Title/Abstract] OR "gut microbiomes"[Title/Abstract] OR "microbiome gut"[Title/Abstract] OR "gut microflora"[Title/Abstract] OR "microflora gut"[Title/Abstract] OR "gut microbiota"[Title/Abstract] OR "gut microbiotas"[Title/Abstract] OR "microbiota gut"[Title/Abstract] OR "gastrointestinal flora"[Title/Abstract] OR "flora gastrointestinal"[Title/Abstract] OR "gut flora"[Title/Abstract] OR "flora gut"[Title/Abstract] OR "gastrointestinal microbiota"[Title/Abstract] OR "gastrointestinal microbiotas"[Title/Abstract] OR "microbiota gastrointestinal"[Title/Abstract] OR "gastrointestinal microbial community"[Title/Abstract] OR "gastrointestinal microbial communities"[Title/Abstract] OR (("Microbiota"[MeSH Terms] OR "Microbiota"[All Fields] OR ("Microbial"[All Fields] AND "Community"[All Fields]) OR "microbial community"[All Fields]) AND "Gastrointestinal"[Title/Abstract]) OR "gastrointestinal microflora"[Title/Abstract] OR "microflora gastrointestinal"[Title/Abstract] OR "gastric microbiome"[Title/Abstract] OR "gastric microbiomes"[Title/Abstract] OR "microbiome gastric"[Title/Abstract] OR "intestinal microbiome"[Title/Abstract] OR "intestinal microbiomes"[Title/Abstract] OR "microbiome intestinal"[Title/Abstract] OR "intestinal microbiota"[Title/Abstract] OR "intestinal microbiotas"[Title/Abstract] OR "microbiota intestinal"[Title/Abstract] OR "intestinal microflora"[Title/Abstract] OR "microflora intestinal"[Title/Abstract] OR "intestinal flora"[Title/Abstract] OR "flora intestinal"[Title/Abstract] OR "enteric bacteria"[Title/Abstract] OR "bacteria enteric"[Title/Abstract])) AND ("randomized controlled trial"[Publication Type] OR "randomized"[Title/Abstract] OR "placebo"[Title/Abstract])
