# Supplementary material for: Investigating the causal association between gut microbiota and type 2 diabetes: a meta-analysis and Mendelian randomization
Source: Front Public Health. 2024 Jun 19;12:1342313. doi: 10.3389/fpubh.2024.1342313 (PMC11220316; doi:10.3389/fpubh.2024.1342313)
Supplement: Supplementary file 8 [file Table_8.DOCX]

library(tidyverse)

library(ggplot2)

library(circlize)

library(grid)

library(ComplexHeatmap)

data <- read.csv(choose.files(),header = T)

data[1:5, 1:5]

library(circlize)

col_fun = list(col_1 = colorRamp2(c(0, 0.5, 1),

c("#6779b2", "white", "#d24b15")),

col_2 = colorRamp2(c(0,0.5, 1),

c("#b2cae3", "#2df8b4", "#b2df8a")),

col_3 = colorRamp2(c(0, 0.5, 1),

c("#1aa06c", "#fb9a99", "#a31a1c")),

col_4 = colorRamp2(c(0,0.5, 1),

c("#fdbf6f", "#af7d00", "#aab2d6")),

col_5 = colorRamp2(c(0, 0.5, 1),

c("#Ff007d", "#6a96c6", "#DFCA59"))

)

circos.clear()

if (T) {

pdf("plot.pdf", height = 7, width = 7)

################### 外圈热图 ========================

circos.par("track.height" = 0.1,

"start.degree" = 0,

"gap.degree" = c(5, 5, 5, 5, 30),

"track.margin" = c(0.01, 0.01))

data_1 <- data[,-4]

for (i in 1:6) {

data_tmp <- as.matrix(as.numeric(data_1[, i+2]))

if (i == 1) {

rownames(data_tmp) <- data$ID

}

colnames(data_tmp) <- paste0(colnames(data_tmp), "-p")

if (i < 6) {

circos.heatmap(data_tmp,

split = data$Level,

col = col_fun[[i]],

rownames.side = "outside",

rownames.cex = 0.3,

cluster = F,

cell.border = NA,

track.height = 0.05)

} else {

data_tmp <- as.matrix(as.numeric(data[, 4]))

colnames(data_tmp) <- "IVW-OR"

circos.track(ylim = range(data_tmp[,1]),

panel.fun = function(x, y) {

circos.text(CELL_META$xcenter,

CELL_META$cell.ylim[2] + mm_y(1.5),

CELL_META$sector.index,

cex = 0.5

)

circos.yaxis(labels.cex = 0.3,

at = seq(0.5, 1.5, 0.2),

sector.index = "phylum")

y = data_tmp[,1][CELL_META$subset]

y = y[CELL_META$row_order]

circos.lines(CELL_META$cell.xlim, c(1, 1), lty = "dashed", col = "black")

circos.points(seq_along(y) - 0.5, y,

pch = 16, cex = 0.5,

col = "#63caff")

if(CELL_META$sector.numeric.index == 5) { # the last sector

cn = rev(c("IVW-p", "MR Egger-p", "WM-p", "SM-p","WMODE-p", "IVW-OR"))

n = length(cn)

circos.text(rep(CELL_META$cell.xlim[2], n) + convert_x(8, "mm"),

c(1, seq(3.5, 9.5, 1.5)), cn,

cex = 0.5, adj = 0.5, facing = "inside")

}

},

track.margin = c(0.02, 0.02),

cell.padding = c(0.02, 0, 0.02, 0))

}

}

circos.clear()

dev.off()

}

getwd()

# For legends

# Create a blank plot

plot(0, 0, type = "n", xlab = "", ylab = "", xlim = c(0, 1), ylim = c(0, 1), main = "Blank Plot")

# Define the color ramps and titles for each plot

color_ramps <- list(

list(c(0, 0.5, 1), c("#6779b2", "white", "#d24b15"), "IVW-pvalue"),

list(c(0, 0.5, 1), c("#b2cae3", "#2df8b4", "#b2df8a"), "MR Egger-pvalue"),

list(c(0, 0.5, 1), c("#1aa06c", "#fb9a99", "#a31a1c"), "Weighted median-pvalue"),

list(c(0, 0.5, 1), c("#fdbf6f", "#af7d00", "#aab2d6"), "Simple mode-pvalue"),

list(c(0, 0.5, 1), c("#Ff007d", "#6a96c6", "#DFCA59"), "Weighted mode-pvalue")

)

# Loop through the color ramps and generate the plots

for (i in 1:length(color_ramps)) {

# Generate the PDF file for each plot

pdf(paste0("plot", i, ".pdf"), height = 7, width = 7)

# Retrieve the color ramp and title for the current plot

col_fun <- colorRamp2(color_ramps[[i]][[1]], color_ramps[[i]][[2]])

title <- color_ramps[[i]][[3]]

# Create the legend

lgd <- Legend(col_fun = col_fun, title = title)

# Draw the legend

draw(lgd, test = "only col_fun")

# Close the PDF file

dev.off()

}
